# Effect of anti-biofilm glass–ionomer cement on *Streptococcus mutans* biofilms

**DOI:** 10.1038/ijos.2015.55

**Published:** 2016-04-29

**Authors:** Su-Ping Wang, Yang Ge, Xue-Dong Zhou, Hockin HK Xu, Michael D Weir, Ke-Ke Zhang, Hao-Hao Wang, Matthias Hannig, Stefan Rupf, Qian Li, Lei Cheng

**Affiliations:** 1 State Key Laboratory of Oral Diseases, West China Hospital of Stomatology, Sichuan University, Chengdu, China; 2 Department of Operative Dentistry and Endodontics, West China Hospital of Stomatology, Sichuan University, Chengdu, China; 3 Biomaterials and Tissue Engineering Division, Department of Endodontics, Prosthodontics and Operative Dentistry, University of Maryland Dental School, Baltimore, USA; 4 Clinic of Operative Dentistry, Periodontology and Preventive Dentistry, Saarland University, Homburg/Saar, Germany

**Keywords:** antibacterial properties, dimethylaminododecyl methacrylate, glass–ionomer cement, material performance, *Streptococcus mutans* biofilms

## Abstract

Dental restorative materials with antimicrobial properties can inhibit bacterial colonization, which may result in a reduction of caries at tooth-filling interaction zones. This study aimed to develop antibacterial glass–ionomer cements (GIC) containing a quaternary ammonium monomer (dimethylaminododecyl methacrylate, DMADDM), and to investigate their effect on material performance and antibacterial properties. Different mass fractions (0, 1.1% and 2.2%) of DMADDM were incorporated into the GIC. The flexure strength, surface charge density, surface roughness and fluoride release were tested. A *Streptococcus mutans* biofilm model was used. Exopolysaccharides (EPS) staining was used to analyze the inhibitory effect of DMADDM on the biofilm matrix. In addition, biofilm metabolic activity, lactic acid metabolism and the expression of glucosyltransferase genes *gtfB, gtfC* and *gtfD* were measured. GIC containing 1.1% and 2.2% DMADDM had flexural strengths matching those of the commercial control (*P*>0.1). DMADDM was able to increase the surface charge density but reduced surface roughness (*P*<0.05). The incorporation of 1.1% and 2.2% DMADDM elevated the release of fluoride by the GIC in the first 2 days (*P*<0.05). The novel DMADDM-modified GIC significantly reduced biofilm metabolic activity (*P*<0.05) and decreased lactic acid production (*P*<0.05). The quantitative polymerase chain reaction (qPCR) results showed that the expression of *gtfB, gtfC* and *gtfD* decreased when mass fractions of DMADDM increased (*P*<0.05). EPS staining showed that both the bacteria and EPS in biofilm decreased in the DMADDM groups. The incorporation of DMADDM could modify the properties of GIC to influence the development of *S. mutans* biofilms. In this study, we investigated the interface properties of antibacterial materials for the first time. GIC containing DMADDM can improve material performance and antibacterial properties and may contribute to the better management of secondary caries.

## Introduction

Dental caries are considered one of the most prevalent chronic diseases in humans worldwide.^1, 2^ The etiology of dental caries is considered a disturbance of the micro-ecological balance of dental plaque.^3^ The dental plaque biofilm grows on all surfaces in the oral cavity, including the teeth, mucosa and all inserted materials. Dental restoration materials are widely used in the treatment of dental caries.^4^ However, half of all dental restorations fail within 10 years, mainly due to secondary caries and bulk fracture.^5, 6, 7^ Quaternary ammonium salts (QAS) have been added to dental materials to combat dental caries, especially to control oral biofilms. Methacryloyloxydodecyl pyridinium bromide was the first QAS to be incorporated as an antibacterial monomer into composite resins and dental adhesives.^8^ The antibacterial effects of dental materials containing other QAS have also been investigated, including methacryloxylethylcetylammonium chloride (DMAE-CB),^9^ quaternary ammonium polyethylenimine^10^, quaternary ammonium dimethacrylate^11, 12^ and dimethylaminododecyl methacrylate (DMADDM).^13, 14^

Glass–ionomer cements (GIC) have been thought to have cariostatic properties. The release of fluoride might reduce demineralization, enhance remineralization and inhibit microbial growth.^15^ However, previous studies indicated that the fluoride release from GIC is not sufficiently potent to inhibit bacterial growth or combat bacterial destruction both adjacent to and below fillings. One previous study tried to add antibacterial polyquaternary ammonium salt into GIC to develop a novel anticaries dental material.^16^ GIC have a major role in the so-called atraumatic restorative therapy, which is widely used in developing countries in rural areas. In addition, the use of GIC has been increasing recently in both pediatric and geriatric dentistry. The primary demand of the fillings that are used for extensive carious processes on dentine and root surfaces under wet conditions is not resistance to chewing forces, but rather the sealing and preservation of the remaining tooth substance. In this context, filling materials with biofilm-inhibiting properties are urgently needed. Because DMADDM has proven to be biocompatible, its use in GIC could be promising to improve this restorative material for the above-mentioned purposes. One recent study confirmed that DMADDM concentrations decreased continuously; after 12 h, the values were found to be near zero for both 1.1% and 2.2% DMADDM-containing specimens in water. In saliva, no DMADDM was found in specimens with the various DMADDM concentrations, which indicated DMADDM's existence in the GIC as a non-releasing agent.^17^

Moreover, the antibacterial mechanism of QAS has been reported in previous studies and is widely thought to be contact killing. It appears that QAS materials can cause bacteria lysis by binding to the cell membrane, thereby causing cytoplasmic leakage. When the negatively charged bacterial cell comes in contact with the positively charged (N^+^) sites of the QAS, the charge interactions will disturb the electrical balance of the cell membrane and the bacteria could lyse under its own osmotic pressure,^10, 18^ but the anti-biofilm action model of GIC containing QAS is not clear. Therefore, it is interesting to investigate whether adding DMADDM into GIC would increase the anti-biofilm benefits or whether it might affect fluoride release. It is known that the properties of material surfaces could influence the adhesion of biofilms. Surface roughness is one of the factors that influence the development of dental plaque.^19^ However, no previous studies focused on whether the incorporation of QAS into GIC would change the surface morphology and surface roughness.

Bacteria in dental biofilms produce both lactic acid and exopolysaccharides (EPS). When the balance of biofilm is disturbed, the accumulation of lactic acid will have a key role in the development of dental caries. Matrix constituents, such as EPS, could affect the diffusion of substances in and out of the biofilm, perhaps helping create a diverse range of microenvironments within the biofilm.^20^ Thus, the metabolic activity of bacteria, acid metabolism and EPS metabolism can be used to monitor the micro-ecological balance of dental plaque biofilms.

Thus, in the present study, we incorporated antibacterial DMADDM into GIC and investigated the effects on both material performance and the *Streptococcus mutans* biofilms. Therefore, it was hypothesized that (1) the addition of DMADDM could influence the material performance of GIC, including flexure strength, surface charge density, surface roughness and fluoride releasing; and (2) DMADDM in GIC will inhibit the cell viability, biofilm metabolism and *gtf* expression in *S. mutans* biofilms.

## Materials and methods

### Fabrication of GIC containing DMADDM

DMADDM was synthesized *via* a modified Menschutkin reaction method.^21^ The GIC chosen for the current study was GC Fuji IX (GC Corporation, Tokyo, Japan). The novel material was modified by adding 0, 5% and 10% DMADDM (*m/m*) to the liquid of the GIC while maintaining the original powder/liquid ratio (3.6:1.0, *m/m*). Thus, the final mass fractions of DMADDM in GIC were 0, 1.1% and 2.2%. The specimens for the biofilm experiments were prepared using the cover of a sterile 48-well plate (Costar, Corning, Corning, NY, USA) as a mould, as described in a previous study.^22^ After immersion in distilled water for 24 h, the GIC bars were sterilized in an ethylene oxide sterilizer (Anprolene AN 74i; Andersen, Haw River, NC, USA).

### Mechanical testing

Each GIC bar was manufactured using a plastic spatula on a mixing paper and was loaded into a 25 mm × 2 mm × 2 mm stainless steel split mould. The GIC bars were then clamped for 10 min at room temperature until they hardened. Thereafter, they were carefully taken out of the moulds. Flexural strength was measured using a three-point flexural test with a 20-mm span at a crosshead speed of 1 mm per min on a computer-controlled Universal Testing Machine (5500R; MTS, Cary, NC, USA). The flexural strength of the material was calculated by


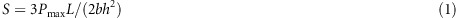


where *P*_max_ is the maximum load on the load–displacement curve, *L* is the flexure span, *b* is the specimen width and *h* is the specimen thickness.

### Charge density

The charge density of the quaternary ammonium groups present on the polymer disk surfaces was quantified using a fluorescein dye method as described previously.^23^ The GIC disks were placed in a 24-well plate. Fluorescein sodium salt (200 μL of 10 mg·mL^−1^) in deionized (DI) water was added to each well, and the specimens were left for 10 min at room temperature in the dark. After removing the fluorescein solution and rinsing extensively with DI water, each sample was placed in a new well, and 200 μL of 0.1% (*m/m*) of cetyltrimethylammonium chloride (CTMAC) in DI water was added. The samples were shaken for 20 min at room temperature in the dark to absorb the bound dye. The CTMAC solution was supplemented with 10% (*V/V*) of 100 mg·mL^−1^ phosphate buffer at pH 8. Sample absorbance was read at 501 nm using a plate reader (SpectraMax M5; Molecular Devices, Sunnyvale, CA, USA). The fluorescein concentration was calculated using Beers Law and an extinction coefficient of 77 mmol·L^−1^·cm^−1^. Using a ratio of 1:1 for fluorescein molecules to the accessible quaternary ammonium groups, the surface charge density was calculated as the total molecules of charge per exposed surface area. Six replicates were tested for each group.

### Atomic force microscope observation

An atomic force microscope (AFM; 5500SPM; Agilent, Santa Clara, CA, USA) was used at high resolution with a sharp silicon tip in tapping mode. The surface topography of the GIC was obtained over an area measuring 20 μm × 20 μm and 5 μm × 5 μm. The surface roughness of the samples was provided by systemic software (SPIWIN 2.0; Seiko, Tokyo, Japan), and the Ra data of different groups were compared.

### Fluoride releasing

Fluoride releasing of the GIC was tested according to previous studies.^24^ Disks (*n*=6) for each group were placed in the wells of a 24-well plate. Each well was inoculated with 1 mL DI water, which was adjusted at pH 5.5 or 7.0. The disks were transferred from the former well to the next every 24 h from the first to the 21st day. The water in each well was collected on days 1–7, 14 and 21 for the measurement of fluoride release, using a fluoride ion selective electrode (Orion star series; Thermo Fisher Scientific, Waltham, MA, USA). Before measurement, 1 mL of Total Ionic Strength Adjustment Buffer II with CDTA (Orion 940909; Thermo Electron, Beverly, MA, USA) was added to each well, and the instrument was calibrated with six standard fluoride solutions containing 0.02 × 10^−6^, 0.2 × 10^−6^, 2.0 × 10^−6^, 20.0 × 10^−6^, 50.0 × 10^−6^ and 100.0 × 10^−6^ F. The final fluoride release results are expressed as μg per cm^2^ per day for further analysis.

### *S. mutans* inoculation and biofilm formation

Fifteen microliters of stock *S. mutans* bacteria (ATCC UA159; American Type Culture Collection, Manassas, VA, USA) was added to 15 mL of brain heart infusion broth (Becton, Dickinson and Company, Franklin Lakes, NJ, USA) and incubated at 37 °C with 5% CO_2_ for 16 h. This *S. mutans* culture was then diluted 10-fold in the growth medium to form the inoculation medium. Each disk was placed in a well of a 24-well plate, inoculated with 1.5 mL of the inoculation medium, and incubated at 5% CO_2_ and 37 °C for 3 days to form mature biofilms.^25^

### Live/dead bacteria staining and EPS staining

After 3 days, the biofilms on the disks were washed three times with PBS and then stained using the BacLight live/dead bacterial viability kit (Molecular Probes, Eugene, OR, USA). The disks were examined using an inverted epifluorescence microscope (Eclipse TE2000-S; Nikon, Melville, NY, USA). An EPS assay was conducted according to previous studies.^26, 27^ In brief, the bacterial cells were labelled with 2.5 μmol·L^−1^ SYTO 9 green fluorescent nucleic acid stain (480 nm/500 nm; Molecular Probes, Eugene, OR, USA). The polysaccharides were labelled with 2.5 μmol·L^−1^ Alexa Fluor 647-dextran conjugate (Thermo Fisher Scientific, Waltham, MA, USA). The disks with 72-h biofilms were examined using confocal laser scanning microscopy (Leica, Wetzlar, Germany).

### 3-(4,5-Dimethylthiazol-2-yl)-2,5-diphenyltetrazolium bromide assays

Disks (*n*=6) with 3-day biofilm in each group were used for the 3-(4,5-dimethylthiazol-2-yl)-2,5-diphenyltetrazolium bromide (MTT) assay. One milliliter of MTT dye (0.5 mg·mL^−1^ MTT in phosphate-buffered saline (PBS)) was added to each well and incubated at 37 °C in 5% CO_2_ for 1 h. After 1 h, the disks were transferred to a new 24-well plate, 1 mL of dimethyl sulfoxide (DMSO) was added to solubilize the formazan crystals and the plate was incubated for 20 min with gentle mixing at room temperature in the dark. After brief mixing *via* pipetting, 200 μL of the DMSO solution from each well was transferred to a 96-well plate, and the absorbance at 540 nm (optical density, OD_540_) was measured *via* the microplate reader.^28^

### Lactic acid measurement

The disks (*n*=6) with 3-day biofilms were rinsed in cysteine peptone water. Each disk was placed in a new 24-well plate, with 1.5 mL of buffered peptone water (BPW) supplemented with 0.2% sucrose. Disks with biofilms were incubated at 5% CO_2_ and 37 °C for 3 h to allow the biofilms to produce acid. Lactate concentrations in the BPW solutions were determined using an enzymatic (lactate dehydrogenase) method.^29^ A microplate reader (SpectraMax M5; Molecular Devices, Sunnyvale, CA, USA) was used to measure the absorbance at 340 nm for the collected BPW solutions. Standard curves were prepared using a standard lactic acid (Supelco Analytical, Bellefonte, PA, USA).

### *S. mutans gtf* gene expression

For the quantitative polymerase chain reaction (qPCR) assays, the biofilms on the disks were collected by centrifugation,^26^ and the RNA was immediately stabilized using the RNA protect Bacteria Reagent (Qiagen, Valencia, CA, USA). Total bacterial RNA isolation was performed using an RNA Bacteria Reagent Kit (Qiagen, Valencia, CA, USA). Purification and reverse transcription were performed using a PrimeScript RT reagent Kit with genomic deoxyribose nucleic acid (gDNA) Eraser (TaKaRa, Shiga, Japan). Real-time PCR was performed for the quantification of *gtfB*, *gtfC* and *gtfD* mRNA expression of the *S. mutans* in the three groups, with 16S rRNA as an internal control. All primers for real-time PCR were obtained commercially from Takara Biotechnology (Dalian, China) and were designed according to methods described in previous studies.^26^ Real-time PCR amplification was performed on the Bio-Rad CFX96 system (Bio-Rad, Hercules, CA, USA). The reaction mixture (25 μL) contained 1 × SYBR green PCR Master Mix (TaKaRa, Shiga, Japan), template cDNA, and forward and reverse primers (10 mmol·L^−1^ each). Threshold cycle values (CT) were determined, and the data were analyzed using Version 2 (Bio-Rad Laboratories, Hercules, CA, USA) according to the 2^-ΔΔCT^ method.

### Statistical analysis

One-way analysis of variance was performed to detect the significant effects of the variables. Tukey's multiple comparison test was used to compare the data at with significance noted at *P*-value of 0.05.

## Results

### Material performance of the novel DMADDM-modified GIC

**Figure 1:**
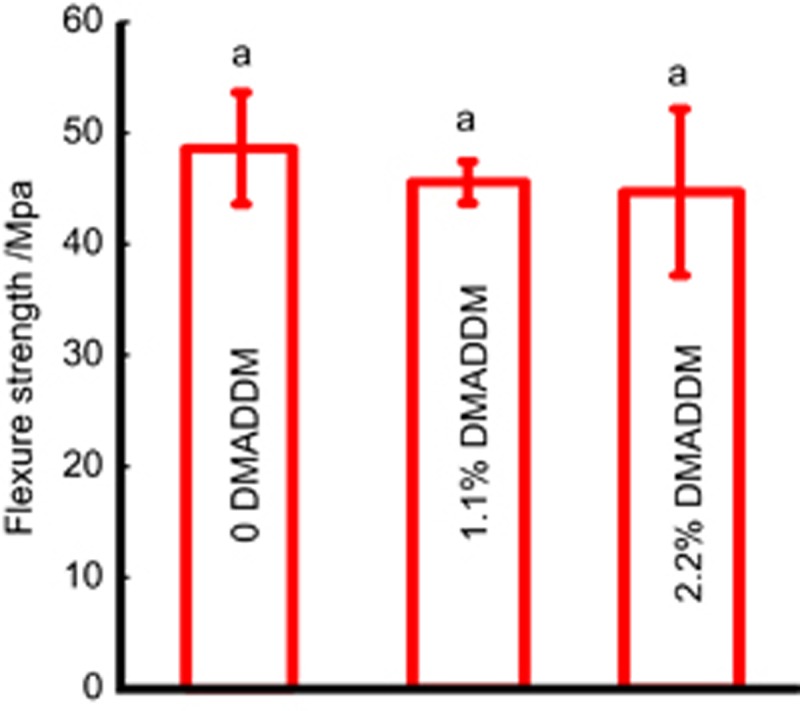
**Mechanical properties.** The flexural strength of GIC containing different mass fractions of DMADDM. Each value is the mean±standard deviation (*n*=6). The three groups had flexural strengths that were not significantly different from each other (*P*<0.05). DMADDM, dimethylaminododecyl methacrylate; GIC, glass–ionomer cements.

The effect of DMADDM on the mechanical properties of GIC is shown in Figure 1. The relationship between different mass fractions and flexural strength was plotted. The results clearly indicate that the GIC with various DMADDM mass fractions (1.1% and 2.2%) had flexural strengths similar to that of the control group.

**Figure 2:**
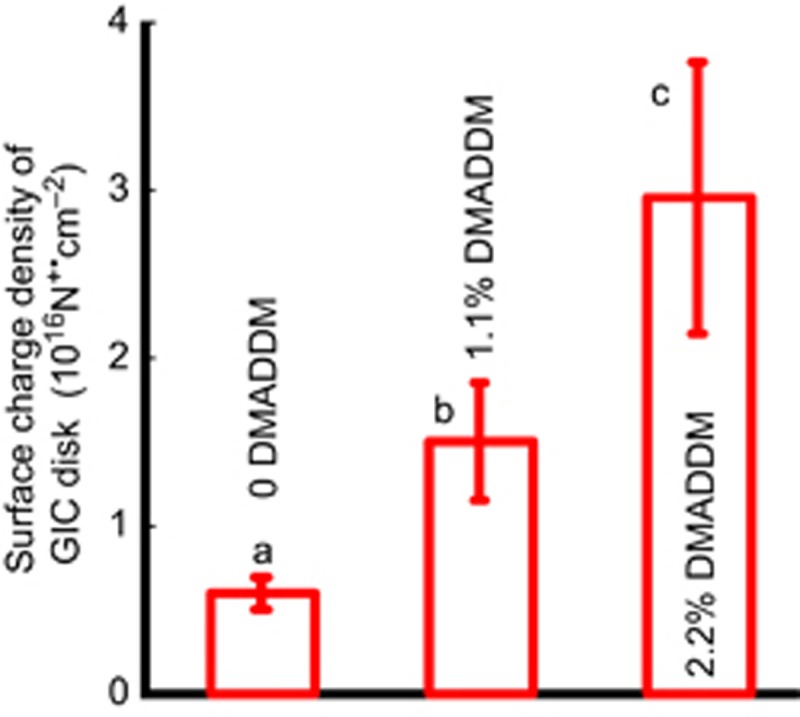
**Surface charge density.** Quaternary amine surface charge densities of the GICs with different mass fractions of DMADDM (mean±standard deviation; *n*=6). The surface charge density significantly increased with increases of the DMADDM mass fraction. Values with dissimilar letters are significantly different from each other (*P*<0.05). DMADDM, dimethylaminododecyl methacrylate; GIC, glass–ionomer cements.

The surface charge density is shown in Figure 2. By adding DMADDM to GIC increased the surface charge density. The charge density value of GIC containing 2.2% DMADDM was about six times that of the control group.

**Figure 3:**
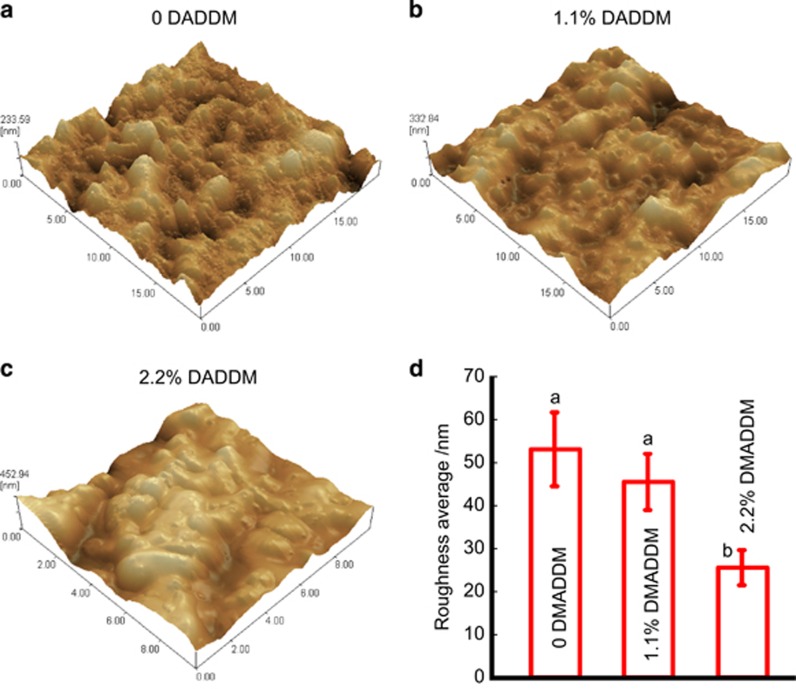
**AFM observation.** (**a**–**c**) Typical AFM images of the surface in the control group, 1.1% DMADDM group and 2.2% DMADDM group. (**d**) The average roughness of GICs containing different mass fractions of DMADDM (mean±standard deviation; *n*=6). Values with dissimilar letters are significantly different from each other (*P*<0.05). AFM, atomic force microscope; DMADDM, dimethylaminododecyl methacrylate; GIC, glass–ionomer cements.

Representative AFM images of the surface in each group are shown in Figure 3a–3c to compare the surface roughness. In Figure 3d, DMADDM decreased the average roughness; GIC containing 2.2% DMADDM had significantly lower roughness values than did the control group.

**Figure 4:**
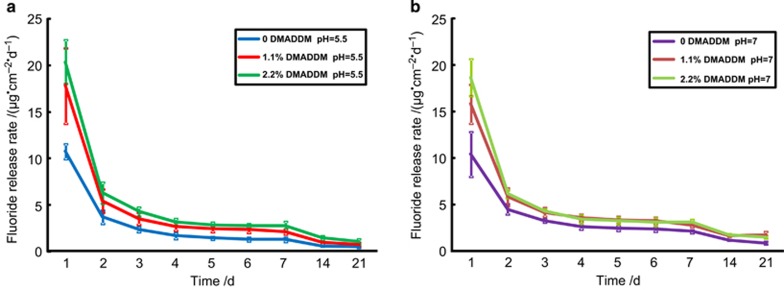
**Fluoride release measurement.** (**a**, **b**) Fluoride release curves of GIC disks with DMADDM after immersion in water at pH 5.5 and pH 7.0 for 21 days. The data represent the means of four independent disks±standard deviation (*P*<0.05). DMADDM, dimethylaminododecyl methacrylate; GIC, glass–ionomer cements.

Figure 4a and 4b plot the fluoride released from the GIC between the 1st and 21st days at different pH. The fluoride release rates decreased sharply in the first 3 days, and then reduced slowly and remained relatively stable from the 3rd to the 21st days in all groups. GIC containing DMADDM (1.1% and 2.2%) released significantly more fluoride than the control group did in the first 2 days (*P*<0.05). After 3 days, the fluoride release trends in the groups were consistent, and the significant difference between the DMADDM groups and the control group persisted (*P*<0.05), except that there was no significant difference between the 0 and 1.1% groups on the 21st day. During the 1st day, the quantity of fluoride released from samples at a pH of 5.5 (Figure 4a) was slightly higher than the quantity released at a pH of 7.0 (Figure 4b), *P*<0.05.

### Antimicrobial properties of the novel DMADDM-modified GIC

**Figure 5:**
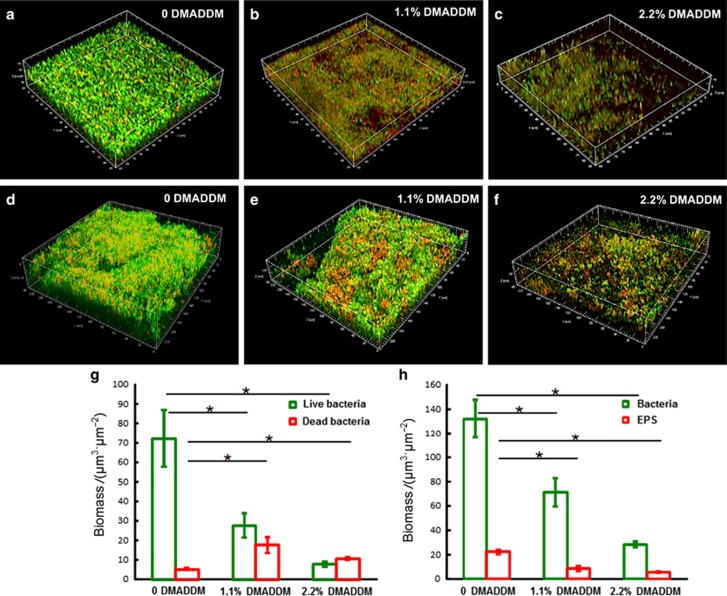
**Live/dead bacteria staining and EPS staining.** (**a**–**c**) Live/dead bacteria staining of the biofilms on the disks of the control group, 1.1% DMADDM group and 2.2% DMADDM group. Living bacteria were stained green and dead bacteria were stained red. Living and dead bacteria in close proximity to each other yielded yellow and orange colours. (**d**–**f**) EPS staining of 72-h biofilms on the disks of the control group, 1.1% DMADDM group and 2.2% DMADDM group. Bacteria were stained green and EPS were stained red. (**g**,**h**) The quantification of bacteria/dead and bacteria/EPS biomass were performed with Imaris 7.0.0. (Olympus, Shanghai, China). The results were averaged from three randomly selected positions of each sample and were presented as the mean±standard deviation (*P*<0.05). DMADDM, dimethylaminododecyl methacrylate; EPS, exopolysaccharides.

Figure 5a–5h show the live/dead bacterial staining and the EPS staining of the biofilms in the different groups. In the control group (Figure 5a), the biofilms were predominantly viable. The GIC containing DMADDM (Figure 5b and 5c) had reduced the biofilm on the surface and increased the proportion of dead bacteria in the biofilm compared with the control group, as shown in Figure 5g. The distributions of bacteria and EPS in the 72-h biofilms in the different groups are shown in Figure 5d–5f. In the 0 DMADDM control group (Figure 5d), more bacteria (green colour) and EPS (red colour) were observed compared with the other groups. Both the bacteria and EPS in the biofilm decreased in the 1.1% DMADDM group (Figure 5e). When 2.2% DMADDM was added into the GIC, the biofilms had fewer bacteria and the EPS was also significantly reduced, as shown in Figure 5f. The adding of DMADDM into GIC could affect the biomass of living bacteria and EPS, whereas the reducing effect obviously decreased as the mass fraction increased in Figure 5h (P<0.05).

**Figure 6:**
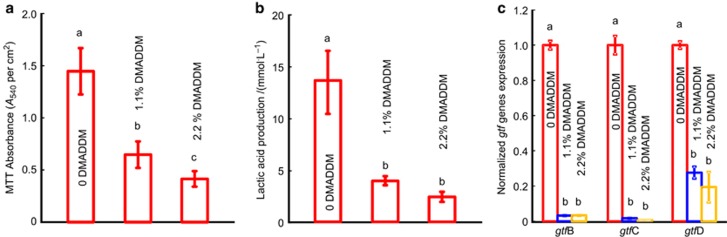
**MTT assays, the lactic acid measurement and real-time PCR.** (**a**) The MTT metabolic activity in the three groups. Each value is the mean±standard deviation (*n*=6). Values with dissimilar letters are significantly different from each other (*P*<0.05). (**b**) Lactic acid production by *S. mutans* biofilms adherent on the disks. Each value is the mean±standard deviation (*n*=6). Dissimilar letters indicate that the values are significantly different from each other (*P*<0.05). (**c**) *S. mutans gtf* gene expression relatively quantified by real-time PCR, with 16S rRNA as an internal control. The results were averaged from three independent experiments and are presented as the mean±standard deviation (*P*<0.05). MTT, 3-(4,5-dimethylthiazol-2-yl)-2,5-diphenyltetrazolium bromide; PCR, polymerase chain reaction.

The MTT assays, lactic acid production and qPCR results are shown in Figure 6a–6c. The MTT results are plotted in Figure 6a. GIC containing 0 DMADDM had the highest absorbance. The GIC containing 1.1% DMADDM had an absorbance 2 times less than that of GIC, and the 2.2% DMADDM group had the lowest absorbance, which was three times less than that of the control group. Lactic acid production is shown in Figure 6b. The biofilm on the disks in control group produced the most acid, followed by those containing 1.1% DMADDM. Acid production on GIC with 2.2% DMADDM was nearly six times less than that on the GIC in the control group. The *gtfB, gtfC* and *gtfD* mRNA expressions are plotted in Figure 6c. Consistent with the reduced EPS production, the glucosyltransferase encoding genes, *gtfB, gtfC* and *gtfD*, of *S. mutans* were also significantly downregulated after adding DMADDM.

## Discussion

The present study investigated both the interface property improvements and the inhibitory effect on *S. mutans* biofilms of the novel GIC containing DMADDM for the first time. The changes in the material performance of the novel GIC are reflected in the surface charge density increase, the reduction of the surface roughness values and the increased fluoride release within the first 2 days, given the premise of its adverse effect on flexure strength. Moreover, GIC with different mass fractions of DMADDM exerted significant anti-biofilm activity compared with the control group, which indicated its favorable antibacterial effects. Increasing the concentration of DMADDM achieved a stronger reduction of biofilm viability, acid production, EPS synthesis and live biofilm volume.

Single-species bacteria biofilms, such as *in vitro* biofilm model systems, are widely used in many scientific setups.^30^ The *S. mutans* biofilm model was chosen for the present study because *S. mutans* biofilms are closely associated with dental caries^31, 32^ and have been used in several previous studies of antibacterial dental materials.^10, 16, 33, 34, 35, 36, 37^ Several previous studies incorporated QAS into the GIC.^16, 38, 39^ Xie *et al.*^16^ developed a novel antibacterial GIC containing polymeric quarternary ammonium salt (PQAS) with a different chain length (CL), and the results indicated that the effects of CL, loading and the grafting ratio of the QAS were significant. In our experiment, we chose DMADDM with CL=12 to add into GIC, which is a novel approach. Previous studies demonstrated that the killing efficacy of QAS was maintained despite the presence of a salivary film.^22, 40^ However, the antibacterial bonding agents with DMADDM had fibroblast/odontoblast cytotoxicities similar to those of the commercial controls.^41^ Preliminary experimental results also found that the added DMADDM releasing concentrations decreased to zero after 12 h for both the 1.1% and 2.2% GIC, as measured by liquid chromatography–mass spectrometry (LC-MS). Which helps ensure its biocompatibility and stable antibacterial properties.

GIC have cariostatic and, to some extent, antibacterial effects owing to the release of fluoride, which is believed to help reduce demineralization, enhance remineralization and inhibit microbial growth.^15^ However, annual clinical surveys found that secondary caries are still the main reason for GIC failure.^42, 43^ In the present study, DMADDM was added to the GIC, which changed the properties of the material's surface, including the surface charge density, average roughness and fluoride release. All of these factors have been proven to potentially influence bacterial adhesion, metabolism and the microecological balance of biofilm. The effects of novel antibacterial GIC on the material properties can be described as follows: increased surface charge density, decreased average surface roughness and increased fluoride release.

As an important aspect of GIC, the mechanical property of GIC containing DMADDM (1.1% and 2.2%) had no adverse effect on flexural strength compared with the commercial non-antibacterial control. Our preliminary experiment demonstrated that a higher mass fraction (3.3%) would reduce its mechanical properties. Considering that this is a short-term and *in vitro* study, further research is needed to ascertain whether the mechanical properties of GIC containing DMADDM are affected by the oral microenvironment and aging.

According to previous studies, the positively charged quaternary amine N^+^ of a QAS was found to attract the negatively charged cell membrane of bacteria, which could disrupt the cell membrane and cause cytoplasmic leakage.^10, 44^ Therefore, the mechanism of positively charged quaternary amine disrupting the negatively charged bacterial membranes could explain the results of the present study, showing that the GIC with a higher density of positive charges had a higher antibacterial power.

Another physical factor that influences bacterial adhesion, that is, the average surface roughness of the GIC, was measured. Notably, the surface roughness decreased with the increase of the mass fraction of DMADDM. Because surface roughness could affect bacterial adhesion,^45^ the decreased surface roughness of the GIC with DMADDM could help reduce the early adhesion of bacteria.

An interesting observation in this experiment is that the incorporation of different mass fractions of DMADDM into the GIC promoted fluoride release compared with the control product, especially in first 2 days. The results indicate that the addition of DMADDM could contribute to the creation of a cariostatic environment around GIC fillings in early stages. The physical presence of DMADDM in the matrix of GIC may help create a pathway for the release of fluoride. It is also possible that the solubility of GIC is increased by adding DMADDM, which may result in the ‘burst' release of fluoride in the early days after filling the defect.

Dental plaque or residual bacteria always colonize the tooth and restoration surfaces, forming biofilms and cariogenic bacteria such as *S. mutans* in the biofilm can metabolize carbohydrates to produce organic acids, which have an important role in the development of secondary caries at the tooth-restoration margins. In the present study, the novel GIC were found to have antibacterial effects on *S. mutans* biofilms. The addition of DMADDM not only helped reduce the biofilm viability but also inhibited acid production and EPS synthesis. In addition, the population of *S. mutans* on the surface of GIC with DMADDM was lower than that of the control group.

*S. mutans* produces at least three genetically separate *gtf*s (*gtfB*, *gtfC* and *gtfD*) genes: *gtfB* synthesizes mostly insoluble glucans rich in α1,3-linked glucose, *gtfC* synthesizes a mixture of insoluble and soluble glucans, and *gtfD* synthesizes predominantly soluble glucans. Consistent with the real-time PCR results, DMADDM-containing GIC suppressed the glucosyltransferases (*gtf*) gene expressions of *S. mutans*, which are important for the synthesis of extracellular glucans and for bacterial cell adhesion and biofilm formation.^20^

There appears to be a connection between the inhibition of biofilm activity, the reduction of lactic acid metabolism and the downregulation of EPS synthesis, and the changes of surface charge density, the average roughness and fluoride release observed in the present study. This could help explain the possible multiple antibacterial mechanisms of the novel GIC *in vitro*. The novel GIC with DMADDM had improved surface properties, the retentive characteristics of fluorine ion release and the remarkable antimicrobial properties of QAS, and it seems to be a ‘bio-active' adhesive material inhibiting biofilm formation and regulating biofilm development in tooth restoration. Because of our use of a single species for the biofilm model in the present experiment, the response of the test organism through manipulation can be monitored as a single variable. Then, in the next step, in explorations of consortium or microcosm biofilms, *in vivo* studies will be needed to test the anti-biofilm effect of DMADDM.

## Conclusions

In conclusion, the current study investigated the interface material properties of antibacterial materials apart from its inhibition on *S. mutans* biofilms for the first time. Given the constant changes of the oral environment, GIC containing DMADDM has improved material performance and antibacterial properties, and may contribute to the improved management of secondary caries.

## References

[bib1] Selwitz RH, Ismail AI, Pitts NB. Dental caries. Lancet 2007; 369 (9555): 51–59. 17208642 10.1016/S0140-6736(07)60031-2

[bib2] Hu DY, Hong X, Li X. Oral health in China—trends and challenges. Int J Oral Sci 2011; 3 (1): 7–12. 21449210 10.4248/IJOS11006 PMC3469869

[bib3] Marsh PD. Dental plaque as a biofilm and a microbial community - implications for health and disease. BMC Oral Health 2006; 6 (Suppl 1): S14. 16934115 10.1186/1472-6831-6-S1-S14 PMC2147593

[bib4] Ferracane JL. Resin composite—state of the art. Dent Mater 2011; 27: 29–38. 21093034 10.1016/j.dental.2010.10.020

[bib5] Spinell T, Schedle A, Watts DC. Polymerization shrinkage kinetics of dimethacrylate resin-cements. Dent Mater 2009; 25 (8): 1058–1066. 19481245 10.1016/j.dental.2009.04.008

[bib6] Drummond JL. Degradation, fatigue, and failure of resin dental composite materials. J Dent Res 2008; 87 (8): 710–719. 18650540 10.1177/154405910808700802 PMC2561305

[bib7] Coelho-De-Souza FH, Camacho GB, Demarco FF et al. Fracture resistance and gap formation of MOD restorations: influence of restorative technique, bevel preparation and water storage. Oper Dent 2008; 33 (1): 37–43. 18335731 10.2341/07-27

[bib8] Imazato S, Kinomoto Y, Tarumi H et al. Antibacterial activity and bonding characteristics of an adhesive resin containing antibacterial monomer MDPB. Dent Mater 2003; 19 (4): 313–319. 12686296 10.1016/s0109-5641(02)00060-x

[bib9] Li F, Chai ZG, Sun MN et al. Anti-biofilm effect of dental adhesive with cationic monomer. J Dent Res 2009; 88 (4): 372–376. 19407160 10.1177/0022034509334499

[bib10] Beyth N, Yudovin-Farber I, Bahir R et al. Antibacterial activity of dental composites containing quaternary ammonium polyethylenimine nanoparticles against *Streptococcus mutans*. Biomaterials 2006; 27 (21): 3995–4002. 16564083 10.1016/j.biomaterials.2006.03.003

[bib11] Cheng L, Zhang K, Melo MA et al. Anti-biofilm dentin primer with quaternary ammonium and silver nanoparticles. J Dent Res 2012; 91 (6): 598–604. 22492276 10.1177/0022034512444128 PMC3348066

[bib12] Cheng L, Weir MD, Zhang K et al. Antibacterial nanocomposite with calcium phosphate and quaternary ammonium. J Dent Res 2012; 91 (5): 460–466. 22403412 10.1177/0022034512440579 PMC3327730

[bib13] Zhou C, Weir MD, Zhang K et al. Synthesis of new antibacterial quaternary ammonium monomer for incorporation into CaP nanocomposite. Dent Mater 2013; 29 (8): 859–870. 23768794 10.1016/j.dental.2013.05.005 PMC3845440

[bib14] Zhou H, Weir MD, Antonucci JM et al. Evaluation of three-dimensional biofilms on antibacterial bonding agents containing novel quaternary ammonium methacrylates. Int J Oral Sci 2014; 6 (2): 77–86. 24722581 10.1038/ijos.2014.18 PMC4071290

[bib15] Wiegand A, Buchalla W, Attin T. Review on fluoride-releasing restorative materials—fluoride release and uptake characteristics, antibacterial activity and influence on caries formation. Dent Mater 2007; 23 (3): 343–362. 16616773 10.1016/j.dental.2006.01.022

[bib16] Xie D, Weng Y, Guo X et al. Preparation and evaluation of a novel glass-ionomer cement with antibacterial functions. Dent Mater 2011; 27 (5): 487–496. 21388668 10.1016/j.dental.2011.02.006

[bib17] Feng J, Cheng L, Zhou X et al. *In situ* antibiofilm effect of glass-ionomer cement containing dimethylaminododecyl methacrylate. Dent Mater 2015; 31 (8): 992–1002. 26059241 10.1016/j.dental.2015.05.005

[bib18] Mei L, Ren Y, Loontjens TJ et al. Contact-killing of adhering streptococci by a quaternary ammonium compound incorporated in an acrylic resin. Int J Artif Organs 2012; 35 (10): 854–863. 23065883 10.5301/ijao.5000149

[bib19] Teughels W, Van Assche N, Sliepen I et al. Effect of material characteristics and/or surface topography on biofilm development. Clin Oral Implants Res 2006; 17 (Suppl 2): 68–81. 16968383 10.1111/j.1600-0501.2006.01353.x

[bib20] Koo H, Falsetta ML, Klein MI. The exopolysaccharide matrix: a virulence determinant of cariogenic biofilm. J Dent Res 2013; 92 (12): 1065–1073. 24045647 10.1177/0022034513504218 PMC3834652

[bib21] Li F, Weir MD, Fouad AF et al. Effect of salivary pellicle on antibacterial activity of novel antibacterial dental adhesives using a dental plaque microcosm biofilm model. Dent Mater 2014; 30 (2): 182–191. 24332270 10.1016/j.dental.2013.11.004 PMC4023513

[bib22] Wang S, Zhang K, Zhou X et al. Antibacterial effect of dental adhesive containing dimethylaminododecyl methacrylate on the development of *Streptococcus mutans* biofilm. Int J Mol Sci 2014; 15 (7): 12791–12806. 25046750 10.3390/ijms150712791 PMC4139875

[bib23] Li F, Weir MD, Chen J et al. Effect of charge density of bonding agent containing a new quaternary ammonium methacrylate on antibacterial and bonding properties. Dent Mater 2014; 30 (4): 433–441. 24534376 10.1016/j.dental.2014.01.002 PMC4312702

[bib24] Mousavinasab SM, Meyers I. Fluoride release by glass ionomer cements, compomer and giomer. Dent Res J (Isfahan) 2009; 6 (2): 75–81. 21528035 PMC3075459

[bib25] Cheng L, Exterkate RA, Zhou X et al. Effect of Galla chinensis on growth and metabolism of microcosm biofilms. Caries Res 2011; 45 (2): 87–92. 21346356 10.1159/000324084

[bib26] Zheng X, Zhang K, Zhou X et al. Involvement of gshAB in the interspecies competition within oral biofilm. J Dent Res 2013; 92 (9): 819–824. 23872989 10.1177/0022034513498598

[bib27] Klein MI, Duarte S, Xiao J et al. Structural and molecular basis of the role of starch and sucrose in *Streptococcus mutans* biofilm development. Appl Environ Microbiol 2009; 75 (3): 837–841. 19028906 10.1128/AEM.01299-08 PMC2632160

[bib28] Cheng L, Weir MD, Xu HH et al. Antibacterial and physical properties of calcium-phosphate and calcium-fluoride nanocomposites with chlorhexidine. Dent Mater 2012; 28 (5): 573–583. 22317794 10.1016/j.dental.2012.01.006 PMC3322264

[bib29] van Loveren C, Buijs JF, ten Cate JM. The effect of triclosan toothpaste on enamel demineralization in a bacterial demineralization model. J Antimicrob Chemother 2000; 45 (2): 153–158. 10660496 10.1093/jac/45.2.153

[bib30] McBain AJ. Chapter 4: *In vitro* biofilm models: an overview. Adv Appl Microbiol 2009; 69: 99–132. 19729092 10.1016/S0065-2164(09)69004-3

[bib31] Moye ZD, Zeng L, Burne RA. Fueling the caries process: carbohydrate metabolism and gene regulation by *Streptococcus mutans*. J Oral Microbiol 2014; 6: 24878. 10.3402/jom.v6.24878 PMC4157138 25317251

[bib32] Zhang S. Dental caries and vaccination strategy against the major cariogenic pathogen, *Streptococcus mutans*. Curr Pharm Biotechnol 2013; 14 (11): 960–966. 24372246 10.2174/1389201014666131226144339

[bib33] Li F, Chen J, Chai Z et al. Effects of a dental adhesive incorporating antibacterial monomer on the growth, adherence and membrane integrity of *Streptococcus mutans*. J Dent 2009; 37 (4): 289–296. 19185408 10.1016/j.jdent.2008.12.004

[bib34] Hu J, Du X, Huang C et al. Antibacterial and physical properties of EGCG-containing glass ionomer cements. J Dent 2013; 41 (10): 927–934. 23911600 10.1016/j.jdent.2013.07.014

[bib35] Fu D, Pei D, Huang C et al. Effect of desensitising paste containing 8% arginine and calcium carbonate on biofilm formation of *Streptococcus mutans in vitro*. J Dent 2013; 41 (7): 619–627. 23643848 10.1016/j.jdent.2013.04.013

[bib36] Du X, Huang X, Huang C et al. Epigallocatechin-3-gallate (EGCG) enhances the therapeutic activity of a dental adhesive. J Dent 2012; 40 (6): 485–492. 22421091 10.1016/j.jdent.2012.02.013

[bib37] Hiraishi N, Yiu CK, King NM et al. Effect of chlorhexidine incorporation into a self-etching primer on dentine bond strength of a luting cement. J Dent 2010; 38 (6): 496–502. 20298738 10.1016/j.jdent.2010.03.005

[bib38] Weng Y, Guo X, Gregory R et al. A novel antibacterial dental glass-ionomer cement. Eur J Oral Sci 2010; 118 (5): 531–534. 20853548 10.1111/j.1600-0722.2010.00770.x

[bib39] Weng Y, Howard L, Chong VJ et al. A novel furanone-modified antibacterial dental glass ionomer cement. Acta Biomater 2012; 8 (8): 3153–3160. 22554887 10.1016/j.actbio.2012.04.038

[bib40] Asri LATW, Crismaru M, Roest S et al. A shape-adaptive,antibacterial-coating of immobilized quaternary-ammonium compounds tethered on hyperbranched polyurea and its mechanism of action. Adv Funct Mater 2014; 24 (3): 346–355.

[bib41] Li F, Weir MD, Fouad AF et al. Time-kill behaviour against eight bacterial species and cytotoxicity of antibacterial monomers. J Dent 2013; 41 (10): 881–891. 23876930 10.1016/j.jdent.2013.07.006 PMC3845446

[bib42] Mjör IA, Dahl JE, Moorhead JE. Placement and replacement of restorations in primary teeth. Acta Odontol Scand 2002; 60 (1): 25–28. 11902609 10.1080/000163502753471961

[bib43] Forss H, Widström E. Reasons for restorative therapy and the longevity of restorations in adults. Acta Odontol Scand 2004; 62 (2): 82–86. 15198387 10.1080/00016350310008733

[bib44] Namba N, Yoshida Y, Nagaoka N et al. Antibacterial effect of bactericide immobilized in resin matrix. Dent Mater 2009; 25 (4): 424–430. 19019421 10.1016/j.dental.2008.08.012

[bib45] Morgan TD, Wilson M. The effects of surface roughness and type of denture acrylic on biofilm formation by *Streptococcus oralis* in a constant depth film fermentor. J Appl Microbiol 2001; 91 (1): 47–53. 11442713 10.1046/j.1365-2672.2001.01338.x

